# High-Grade Undifferentiated Pleomorphic Sarcoma of the Distal Femur Masquerading as a Medial Collateral Ligament Sprain in a Recreational Runner: A Case Report

**DOI:** 10.7759/cureus.117008

**Published:** 2026-09-27

**Authors:** Nina Reddy, Vahini Srikakulapu, David A Ross

**Affiliations:** 1 Herbert Wertheim College of Medicine, Florida International University, Miami, USA; 2 Sports Medicine, Ross Medical Group, Miami, USA

**Keywords:** bone tumor, knee pain, pathologic fracture, sports medicine, undifferentiated pleomorphic sarcoma

## Abstract

A 62-year-old recreational runner with osteopenia presented to a sports medicine clinic with left medial knee pain following a non-contact hyperextension injury sustained during a training run. Physical examination demonstrated medial joint line tenderness with positive valgus and varus stress testing, indicating laxity of both collateral ligaments that, in retrospect, was inconsistent with an isolated medial collateral ligament (MCL) injury and out of proportion to the low-energy mechanism. Initial radiographs revealed a moth-eaten pattern in the distal femoral meta-diaphysis. MRI demonstrated a 4.4 × 6.6 × 9 cm mass in the left distal femoral meta-diaphysis with cortical breakthrough and soft-tissue extension. Ultrasound-guided biopsy confirmed high-grade undifferentiated pleomorphic sarcoma of bone (UPS-B), staged T2NxM1a (Stage IVA). While awaiting pathology results, the patient sustained a displaced pathologic fracture through the lesion. She had an inadequate response to neoadjuvant chemotherapy and ultimately underwent an above-knee amputation. UPS-B is a rare primary malignant bone tumor that can present with symptoms mimicking common sports injuries. This case highlights the importance of recognizing an inconclusive examination and obtaining radiographs in older active adults with atypical musculoskeletal presentations.

## Introduction

Primary malignant bone tumors can present with symptoms that closely mimic common sports injuries, creating significant potential for diagnostic delay [1-3]. Tumors about the knee are especially prone to being mistaken for athletic injuries, and such misdiagnosis can adversely affect oncologic outcomes [2]. In an older, physically active patient, new knee pain after a minor mechanical event is easily attributed to a soft-tissue sports injury, which can delay recognition of an underlying bone malignancy.

Undifferentiated pleomorphic sarcoma of bone (UPS-B), formerly known as malignant fibrous histiocytoma of bone, is an exceedingly rare primary malignant bone tumor that accounts for less than 1% of all bone sarcomas [4-6]. Unlike osteosarcoma, which mainly affects adolescents and young adults, UPS-B tends to occur in older patients, with a median age at diagnosis of roughly 42 to 55 years [4-6]. It has no specific line of differentiation and is diagnosed by exclusion after immunohistochemical and molecular workup [7]. Because it favors older adults, its presentation is especially easy to mistake for a routine degenerative or sports-related complaint.

We report a case of high-grade UPS-B in a 62-year-old recreational runner, which initially presented as medial knee pain after a low-energy, non-contact injury. The main red flag was an inconclusive knee examination: both valgus and varus stress testing were positive, a pattern that an isolated collateral ligament injury does not explain. That finding, along with focal bony tenderness and a course that worsened rather than improved, is what prompted plain radiography. There is no established set of examination features that reliably separates early neoplastic knee pain from a benign sports injury, and this case illustrates one such examinable warning sign. The patient later sustained a pathologic fracture through the lesion during the workup, underscoring the structural fragility these tumors can produce.

## Case presentation

A 62-year-old woman with a history of osteopenia, vitamin D deficiency, and thiamine deficiency presented with mild left medial knee pain after a non-contact hyperextension injury sustained while stepping off a curb during a training run for a 5-kilometer race. She was able to continue her run but noted that the medial pain worsened over the following days rather than improving. She denied swelling, instability, systemic symptoms, night pain, or unintentional weight loss. Her family history was unremarkable, with no known bone or soft-tissue malignancy. Table 1 summarizes the clinical timeline and key findings.

**Table 1:** Clinical timeline and key clinical, imaging, and pathology findings Intervals are given in days relative to the injury (day 0). ROM, range of motion; MCL, medial collateral ligament; IHC, immunohistochemistry; AIM: Adriamycin/doxorubicin, ifosfamide, and mesna

Timepoint	Event/Key Findings
Day 0	Non-contact hyperextension injury stepping off a curb during a training run; able to continue running, with medial knee pain that then worsened.
Day 5	First clinic visit. Full painless ROM (0–140°); negative anterior drawer, Lachman, McMurray; positive valgus and varus stress testing; tenderness at the medial joint line, MCL, and medial femoral condyle; no external swelling. Radiographs: moth-eaten distal femoral meta-diaphysis with soft-tissue extension.
Day 15	MRI: 4.4 × 6.6 × 9 cm distal femoral meta-diaphyseal mass with cortical breakthrough and soft-tissue extension, mass effect on the vastus medialis.
Day 18	Orthopedic oncology referral. FDG-PET: intense uptake confined to the left distal femur, no distant skeletal uptake. CT chest: scattered bilateral subcentimeter pulmonary nodules (up to 6 mm), indeterminate on the initial read.
Day 30	Ultrasound-guided core needle biopsy and fine-needle aspiration.
Day 32	Displaced pathologic fracture through the lesion during ordinary household ambulation.
Diagnosis	High-grade undifferentiated pleomorphic sarcoma of bone (diagnosis of exclusion). IHC negative for pankeratin, S100, CD34, desmin, myogenin, HMB45, SOX10; partial weak MDM2; patchy strong SATB2; osteosarcoma excluded on imaging grounds. Stage T2NxM1a (Stage IVA).
Treatment	AIM neoadjuvant chemotherapy (doxorubicin, ifosfamide, mesna) × 2 cycles with inadequate response; above-knee amputation (~day 101). Resection margin status and treatment-related necrosis not available at this time.
Follow-up	Monthly orthopedic oncology, oncology, and sports medicine; enrolled in an investigational systemic therapy trial. Interval CT chest (~3 months post-op): new scattered subcentimeter pulmonary nodules consistent with pulmonary metastatic disease; restaging pending.

On inspection, there was no swelling, effusion, erythema, or ecchymosis. Range of motion (ROM) was full and painless, from 0° to 140°. Anterior drawer, Lachman, and McMurray tests were negative. Both valgus and varus stress testing were positive, indicating laxity of the medial and lateral collateral structures. Palpation elicited tenderness along the medial joint line, MCL, and medial femoral condyle. Laxity in both directions is not what an isolated collateral ligament sprain produces. Following a low-energy, non-contact mechanism, and with no external swelling despite marked medial tenderness, the examination was inconclusive rather than typical of a straightforward MCL injury. No definitive ligamentous diagnosis was made, and no treatment was started before radiographs were obtained at this first visit. Initial anteroposterior and lateral radiographs of the left knee demonstrated a moth-eaten appearance of the distal femoral meta-diaphysis with associated soft-tissue extension, raising concern for an aggressive osseous process (Figure 1). MRI demonstrated a 4.4 × 6.6 × 9 cm mass centered in the left distal femoral meta-diaphysis with cortical breakthrough and soft-tissue extension toward the suprapatellar region, exerting mass effect on the vastus medialis (Figure 2). These findings were concerning for a malignant bone tumor.

**Figure 1:**
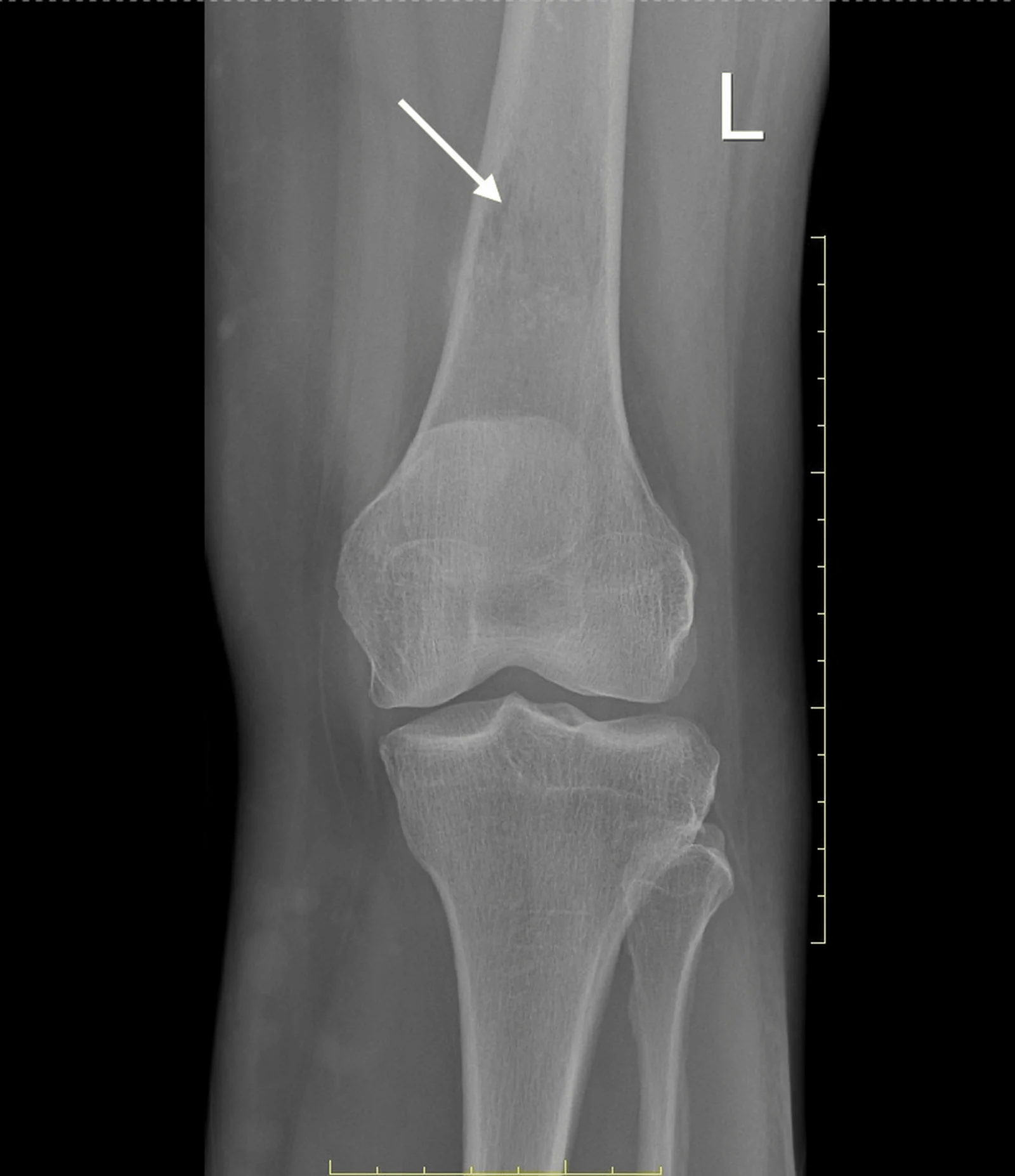
Anteroposterior radiograph of the left knee demonstrating a moth-eaten appearance of the distal femoral meta-diaphysis (arrow) with associated soft-tissue extension, consistent with an aggressive osseous process

**Figure 2:**
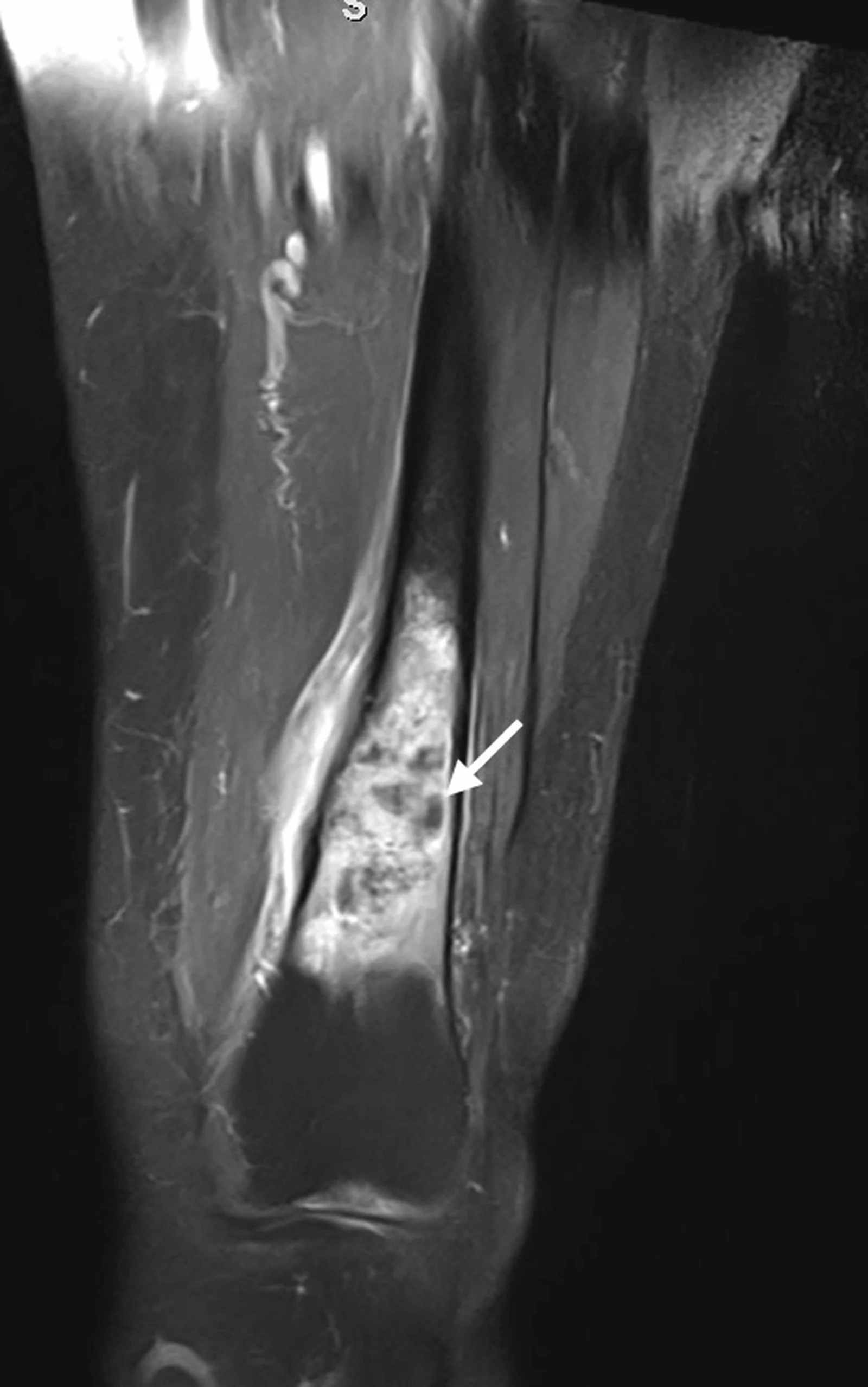
Magnetic resonance image of the left distal femur demonstrating a 4.4 × 6.6 × 9 cm mass centered in the distal femoral meta-diaphysis with cortical breakthrough and soft-tissue extension toward the suprapatellar region, exerting mass effect on the vastus medialis (arrow)

The patient was urgently referred to orthopedic oncology. Fluorodeoxyglucose-positron emission tomography (FDG-PET) demonstrated intense uptake confined to the left distal femur without distant skeletal uptake (Figure 3). CT of the chest revealed scattered bilateral subcentimeter pulmonary nodules (up to 6 mm) that were indeterminate on the initial read (Figure 4). Taking the pulmonary findings into account, the tumor was staged T2NxM1a (Stage IVA). Ultrasound-guided core needle biopsy and fine-needle aspiration of the distal femoral lesion were performed.

**Figure 3:**
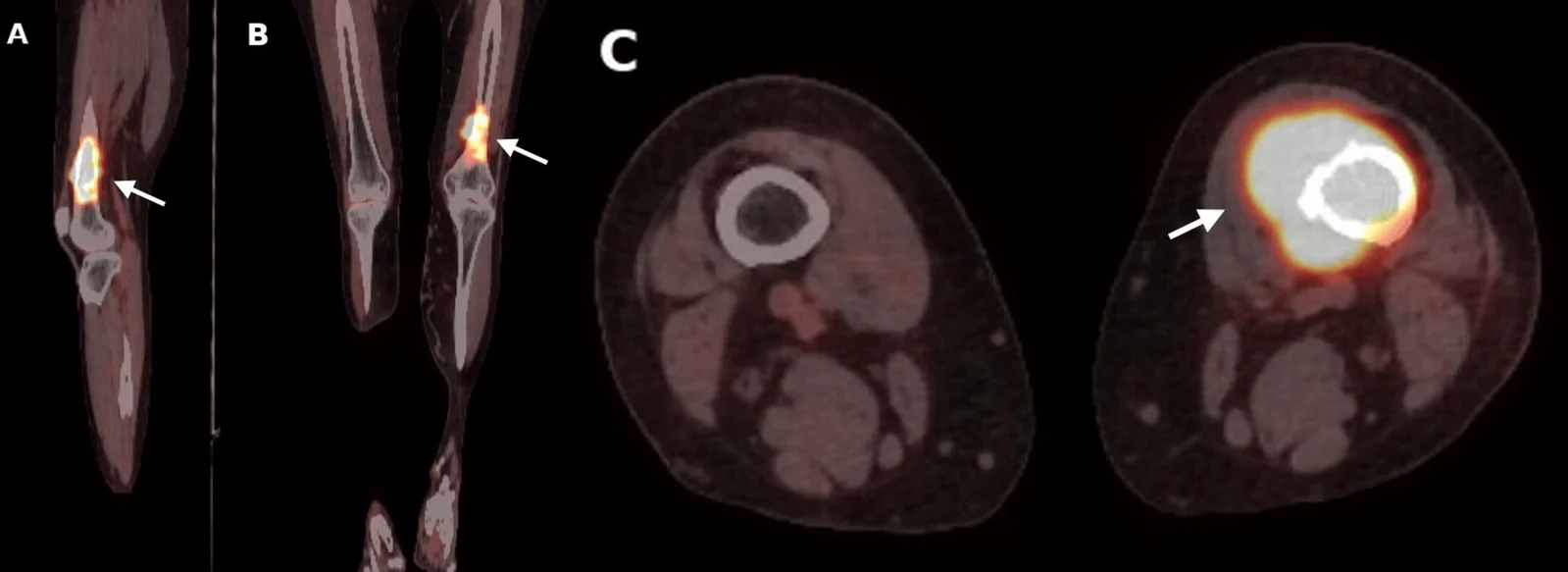
FDG-PET of the left lower extremity Sagittal (A), coronal (B), and axial (C) fused fluorodeoxyglucose positron emission tomography (FDG-PET) images demonstrate intense FDG uptake confined to the left distal femur (arrow), without distant skeletal uptake. On the axial image (C), the uptake is seen in the left thigh (image right).

**Figure 4:**
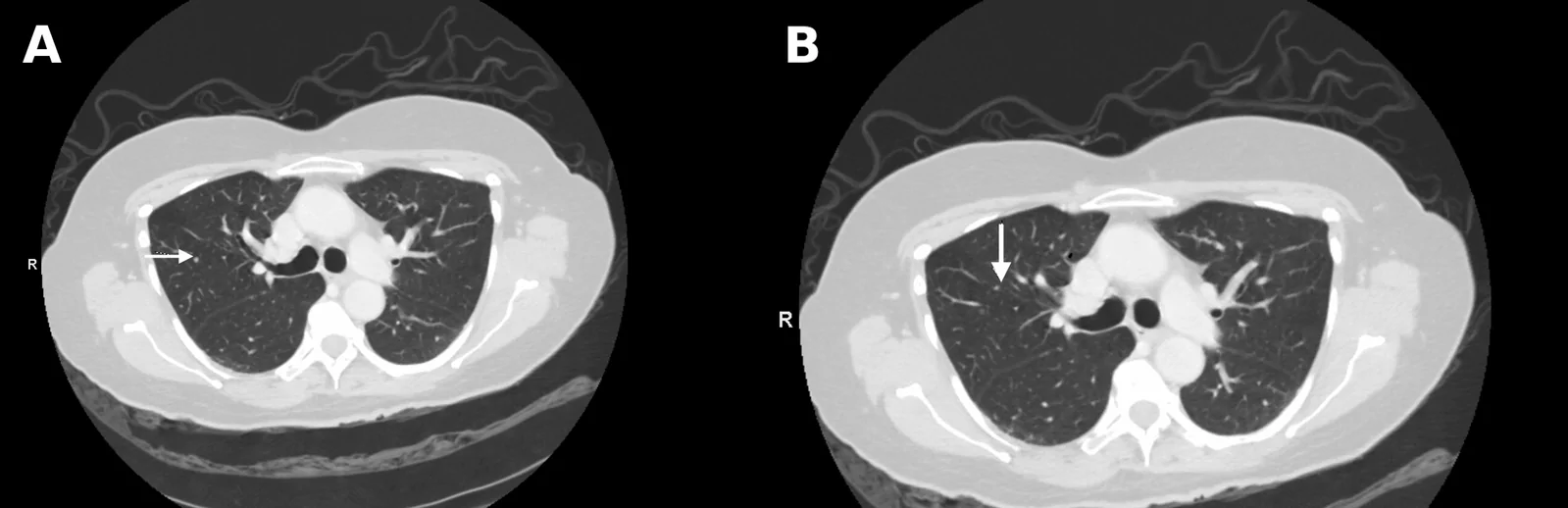
CT of the chest Axial computed tomography (CT) images of the chest in lung windows demonstrate scattered bilateral pulmonary nodules measuring up to 6 mm (arrows), of indeterminate significance.

Two days after the biopsy, while awaiting final pathology, the patient sustained a displaced pathologic fracture of the left distal femur through the lesion during ordinary household ambulation, before any definitive surgical stabilization (Figure 5). MRI further characterized the fracture and the associated soft-tissue mass (Figure 6). Histopathologic examination of the core biopsy and aspiration revealed a high-grade spindle and pleomorphic cell neoplasm without identifiable osteoid production, chondroid matrix, or specific line of differentiation. On immunohistochemistry, the lesion was negative for pankeratin, S100, CD34, desmin, myogenin, HMB45, and SOX10, arguing against carcinoma, melanoma, nerve sheath, and skeletal-muscle differentiation. There was partial weak MDM2 expression and patchy strong SATB2 staining. Although SATB2 can be seen in osteosarcoma, it is also reported in a substantial proportion of bone UPS, and the imaging did not support osteosarcoma. Combining the immunohistochemical profile with the imaging findings, the final diagnosis was high-grade undifferentiated pleomorphic sarcoma of bone, reached as a diagnosis of exclusion. Because immunohistochemistry can give false-negative results on decalcified tissue, the negative stains were read with that caveat in mind.

**Figure 5:**
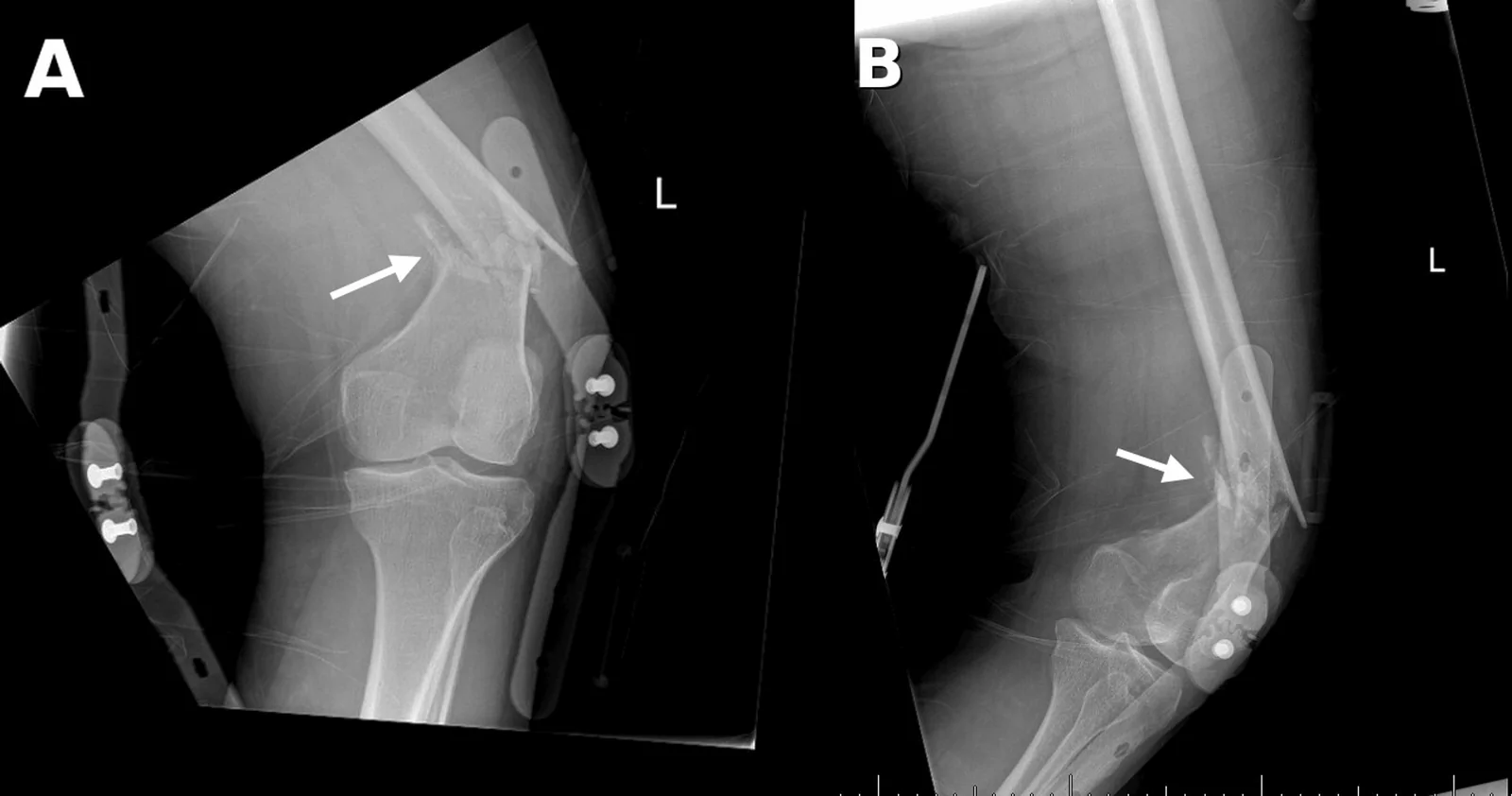
Radiographs of the pathologic fracture Anteroposterior (A) and lateral (B) radiographs of the left femur demonstrate a displaced pathologic fracture (arrow) through the underlying lytic lesion.

**Figure 6:**
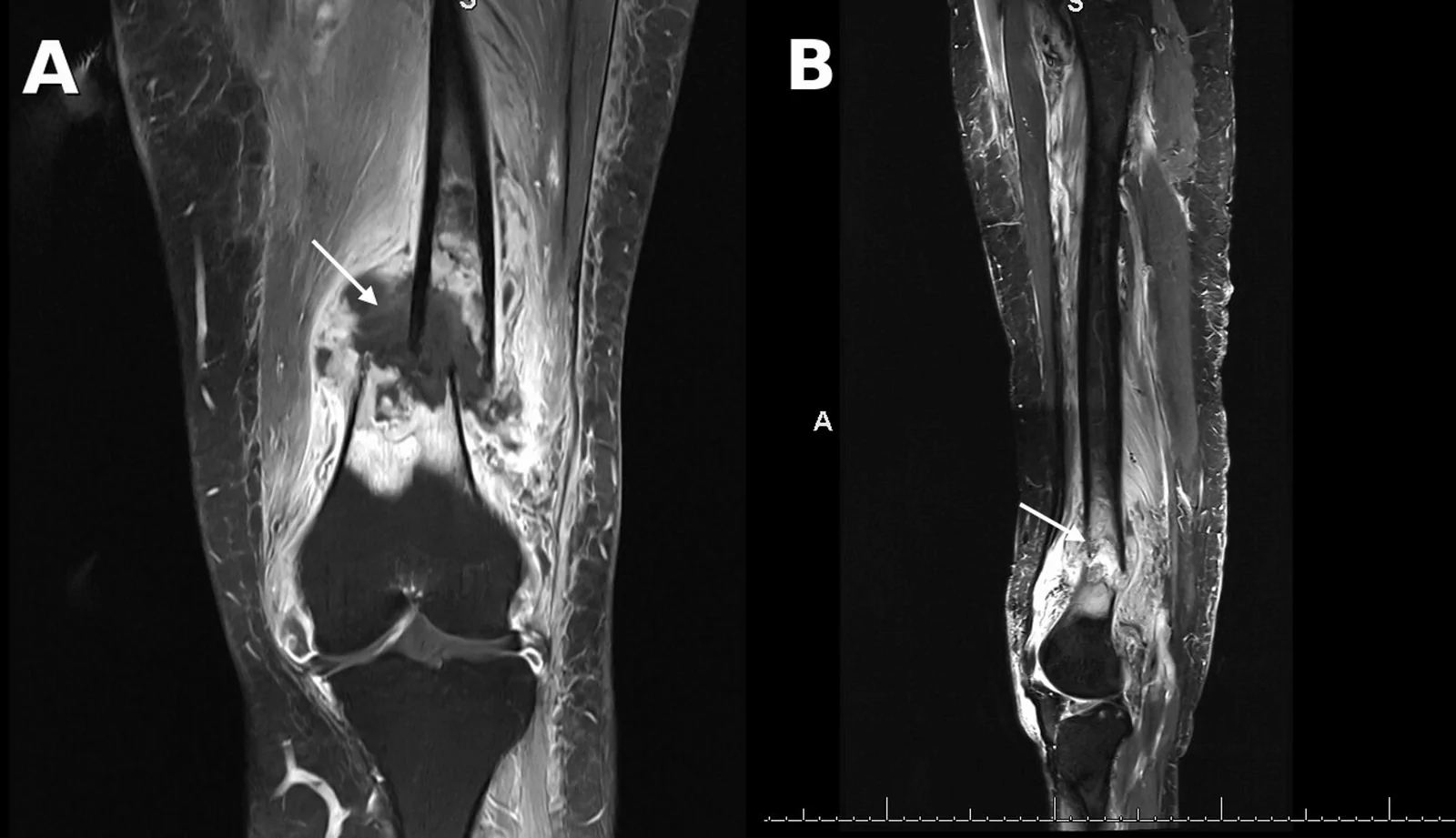
MRI of the pathologic fracture Coronal (A) and sagittal (B) magnetic resonance imaging (MRI) images demonstrate the pathologic fracture through the distal femoral lesion with an associated soft-tissue mass (arrow).

The pathologic fracture was managed with closed reduction, long-leg casting, and Buck’s traction. Orthopedic oncology advised against internal fixation given the extent of disease. Following multidisciplinary tumor board discussion, the patient began AIM neoadjuvant chemotherapy (Adriamycin/doxorubicin, ifosfamide, and mesna). After two cycles with an inadequate response, and given a large meta-diaphyseal tumor with cortical breakthrough, soft-tissue extension, and a displaced pathologic fracture through the lesion, limb-sparing resection was not felt to be feasible, and she underwent an above-knee amputation. Resection margin status and treatment-related necrosis from the amputation specimen are not available at this time; the present report focuses on the initial diagnosis and the diagnostic pathway. She was fitted for a custom prosthesis and has since been followed monthly by orthopedic oncology, oncology, and sports medicine, and is enrolled in an investigational systemic therapy trial. Interval CT of the chest about three months after surgery showed new scattered subcentimeter pulmonary nodules consistent with pulmonary metastatic disease, with restaging pending. Return to exercise remains pending response to systemic therapy.

## Discussion

Because UPS-B is a diagnosis of exclusion, the diagnosis rests on integrating immunohistochemistry, imaging, and, where needed, molecular studies [7]. In this case, a broad immunohistochemical panel excluded carcinoma, melanoma, nerve sheath, and skeletal-muscle lineages, and osteosarcoma was excluded on combined pathologic and imaging grounds.

The clinical presentation of primary bone sarcomas is frequently nonspecific, resulting in diagnostic delay. Widhe and Widhe reported that the most common misdiagnosis for osteosarcoma was tendinitis (31%) and that 47% of patients related symptom onset to minor trauma [8]. Wurtz et al. found that 44% of patients with primary pelvic bone sarcomas were initially misdiagnosed with benign musculoskeletal conditions [9]. Delayed diagnosis is the primary cause of sarcoma-related malpractice litigation in the United States, cited in 91% of claims [1].

Tumors about the knee are particularly susceptible to misdiagnosis as athletic injuries. Muscolo et al. reported that 3.7% of knee tumors had previously been treated with intra-articular procedures based on an erroneous sports injury diagnosis, adversely affecting oncologic treatment in 60% of those cases [2]. Krych et al. emphasized that pain unrelated to activity and a clinical course deviating from the expected recovery trajectory should raise suspicion for an underlying neoplastic process [3].

A key feature in this case was an inconclusive knee examination. Both valgus and varus stress testing were positive, indicating laxity of the medial and lateral collateral structures. An isolated MCL sprain would produce valgus laxity alone; the added varus laxity pointed to instability beyond a single ligament and did not fit an isolated MCL injury, particularly after a low-energy, non-contact mechanism. Multidirectional laxity is not specific to a tumor and can follow other capsuloligamentous injury, so the useful point is simply that the examination did not cohere with a single-ligament sprain. Taken with focal tenderness over the medial femoral condyle and a course that worsened rather than improved, these findings raised concern for an underlying osseous process and supported obtaining plain radiographs.

The clinical lesson rests on the combination of findings rather than any single one: an inconclusive examination, the moth-eaten radiographic pattern, cortical breakthrough, and the later pathologic fracture. In this case, radiographs identified the moth-eaten osseous pattern and immediately raised concern for an aggressive lesion, while MRI defined the extent of disease and guided biopsy planning and orthopedic oncology referral. The largest series of UPS-B to date, the COSS report of 132 patients, found extremity involvement in 86% of cases and pathologic fractures in 14% of extremity primaries [5]. In the present case, the patient sustained a pathologic fracture while awaiting biopsy results, illustrating how structural fragility from cortical invasion can precede definitive diagnosis.

## Conclusions

This case highlights several features that, taken together, may help sports medicine practitioners evaluate knee pain in older active adults: an inconclusive or internally inconsistent examination, pain that worsens rather than improves after a low-energy injury, advanced age, and a clinical course that does not follow the expected recovery trajectory. Although these observations come from a single case, they are supported by prior series on missed and delayed diagnoses of sarcoma. When these features are present, early plain radiography, reviewed with an appropriate index of suspicion for osseous pathology, is a reasonable next step. If radiographs are abnormal or equivocal, MRI should follow to characterize the extent of the lesion and expedite orthopedic oncology referral.
